# Rare occurrence of common filaggrin mutations in Turkish children with food allergy and atopic dermatitis

**DOI:** 10.3906/sag-1910-162

**Published:** 2020-12-17

**Authors:** Neşe VARDAR ACAR, Özlem CAVKAYTAR, Ebru ARIK YILMAZ, Betül BÜYÜKTİRYAKİ, Özge SOYER, Ümit Murat SAHİNER, Bülent Enis ŞEKEREL, Çağatay KARAASLAN, Cansın SAÇKESEN

**Affiliations:** 1 Department of Biology, Faculty of Science, Hacettepe University Ankara Turkey; 2 Department of Pediatric Allergy, Faculty of Medicine, Hacettepe University, Ankara Turkey; 3 Department of Pediatric Allergy and Immunology, Faculty of Medicine, İstanbul Medeniyet University, İstanbul Turkey; 4 Department of Pediatric Allergy, Faculty of Medicine, Pamukkale University, Denizli Turkey; 5 Department of Pediatric Allergy, Faculty of Medicine, Koç University, İstanbul Turkey

**Keywords:** Atopic dermatitis, filaggrin, food allergy, R501X, 2282del4, R2447X, S3247X

## Abstract

**Background/aim:**

Filaggrin is a protein complex involved in epidermal differentiation and skin barrier formation. Mutations of the filaggrin gene (FLG) are associated with allergen sensitization and allergic diseases like atopic dermatitis (AD), allergic rhinitis, food allergy (FA), and asthma. The aim of the study is to reveal the frequency of change in the FLG gene and determine the association between FLG loss-of-function (LOF) mutations and FA and/or AD in Turkish children.

**Materials and methods:**

Four *FLG* loss-of-function (LOF) mutations known to be common in European populations were analyzed in 128 healthy children, 405 food-allergic children with or without atopic dermatitis, and 61 children with atopic dermatitis. PCR-RFLP was performed for genotyping R501X, 2282del4, and R2447X mutations; S3247X was genotyped using a TaqMan-based allelic discrimination assay. Results were confirmed by DNA sequence analysis in 50 randomly chosen patients for all mutations.

**Results:**

A total of 466 patients [(67% male, 1 (0.7–2.8) years] and 128 healthy controls [59% male, 2.4 (1.4–3.5) years)] were included in this study. Two patients were heterozygous carriers of wild-type R501X, but none of the controls carried this mutation. Three patients and one healthy control were heterozygous carriers of wild-type 2282del4. Neither patients nor controls carried R2447X or S3247X *FLG* mutations. There were no combined mutations determined in heterozygous mutation carriers.

**Conclusions:**

Although R501X, 2282del4, R2447X, and S3247X mutations are very common in European populations, we found that FLG mutations were infrequent and there is no significant association with food allergy and/or atopic dermatitis in Turkish individuals.

## 1. Introduction

Filaggrin (FLG) is a protein complex involved in epidermal differentiation and skin barrier formation [1]. Filaggrin is first synthesized as profilaggrin in the keratohyalin granules of the stratum granulosum [2]. During the terminal differentiation of keratinocytes, profilaggrin is activated by dephosphorylation and proteolytic cleavage. Then, FLG monomers aggregate in the keratin filament as tight bundles causing the collapse of the keratinocytes, which become flattened and tightly packed and form the stratum corneum (SC) [1,2]. Filaggrin monomers in the SC are deiminated and degraded into amino acids including histidine, arginine, and glutamine which are then hydrolyzed to form natural moisturizing factors (NMFs) which mainly contribute to epithelial hydration and the barrier function of the SC [3]. In addition to the moisture retention function of NMFs, they perform functions essential to acidic pH maintenance and inhibition of pathogenic bacteria [3,4].

Previous studies have shown that loss-of-function (LOF) mutations in the FLG gene are associated with allergic diseases such as atopic dermatitis (AD), allergic rhinitis, food allergy (FA), and asthma [5]. Filaggrin mutation has been identified as an important risk factor, particularly for the development of AD, and LOF mutations in FLG are found in 16%–44% of European patients with moderate to severe AD [4]. The spectrum of mutations in FLG show a population-specific distribution profile among Asian, African American, and Northern and Southern European patients [6]. Although over 40 LOF mutations of FLG were determined in European and Asian populations to date, a limited number of them, such as R501X, 2282del4, R2447X, and S3247X mutations, are common in European Caucasians [1,4,6].

In a mouse model a 1-base pair deletion mutation in FLG, which is analogous to common human FLG mutations, predisposed antigen transfer through a defective epidermal barrier and resulted in elevated food allergen IgE sensitization [7]. Thereafter, the association of sensitization to food allergens, such as hen’s egg, cow’s milk, and peanut, with LOF mutations in the FLG gene has been investigated in many clinical studies, and these studies exhibit some contradictory results [7–9]. Filaggrin mutations increased the risk of food sensitization in the first year of life but did not play a further role in progression from asymptomatic egg and peanut sensitization to FA [8]. In other studies, having FLG-LOF mutations was strongly associated with clinical FA [10,11].

The aim of this study was to determine the frequency of the most common LOF mutations in a Turkish population and to investigate the association of FLG-LOF mutations with FA and AD.

## 2. Materials and methods

### 2.1. Study participants

Patients who were referred to Hacettepe University, Division of Pediatric Allergy and diagnosed with IgE-mediated FA (hen’s egg, cow’s milk, tree nuts, peanut, wheat, legumes, beef, etc.) with or without AD and patients who were referred due to AD without a history of FA between January 2009 and May 2013 were included in the study. The serum total and specific IgE levels were measured with the ImmunoCAP system in accordance with manufacturer instructions (Thermo Fisher Scientific, Uppsala, Sweden). Eosinophil counts were identified using the Coulter Counter (Beckman Coulter, California, USA) [12]. IgE-mediated FA diagnosis was based on the presence of a consistent clear-cut history of allergic symptoms that occurred after the ingestion of a specific food together with the presence of either elevated titers of serum specific IgE (>0.35 kU/L) or a positive skin-prick test for the respective food allergen [13]. The diagnosis of AD was based on the presence of chronic pruritus and eczematous dermatitis with typical age-specific morphology, distribution pattern, and a chronic relapsing course [14]. The severity of AD was determined by SCORing atopic dermatitis index (SCORAD) and graded as severe or nonsevere (mild to moderate) AD [15]. Inclusion criteria for patients were a diagnosis of either IgE-mediated FA and/or AD based on the abovementioned criteria. The exclusion criteria include being diagnosed with a chronic systemic illness apart from allergic diseases, such as chronic respiratory, cardiac, gastroenterological, neurologic, or genetic diseases. Patients were divided into three groups: patients with FA and with AD (FA w AD), solely FA without AD (FA w/o AD), and AD without FA (AD w/o FA).

The patients were compared according to demographic and clinical features as well as their baseline eosinophil counts and total IgE values. Diagnoses of asthma and allergic rhinitis were based on guidelines [16,17].

Age- and sex-matched healthy controls constituted the control group of study participants. They were recruited from patients who applied to the general pediatrics outpatient clinics of the hospital due to a nonallergic complaint and who were not there for follow-up due to a systemic, chronic illness. The past medical history of healthy controls was taken by questionnaire and given to all control participants and their families to screen for and exclude the presence of any allergic disease, including allergic rhinitis, atopic dermatitis, asthma, food allergy, chronic urticaria, and venom hypersensitivity. Four common FLG-LOF mutations in European populations (R501X, 2282del4, R2447X, and S3247) were screened for by PCR-RFLP and the TaqMan genotyping method. Results were confirmed by DNA sequence analysis in 50 randomly chosen patients for all mutations.

### 2.2. Genotyping of filaggrin gene mutations

Genomic DNA was extracted from whole blood samples using the modified methods of Poncz et al. [18]. Four common FLG-LOF mutations in European populations (R501X, 2282del4, R2447X, and S3247) were screened for by PCR-RFLP and TaqMan genotyping method [19,20]. PCR-RFLP was performed for genotyping R501X, 2282del4, and R2447X mutations; S3247X was genotyped by TaqMan-based allelic discrimination assay (Applied Biosystems, Foster City, CA, USA). PCR primers and wild and mutant probe sequences are listed in Table 1.

**Table 1:** Primers and probes for genotyping of the FLG mutations.

R501	Forward 5’-CACGGAAAGGCTGGGCTG-3’
	Reverse 5’-ACCTGAGTGTCCAGACCTATT-3’
2282del4	Forward 5’-ATTAGGTCTGGACACTCAGGT-3’
	Reverse 5’-GGGAGGACTCAGACTGTTT-3’
R2447X	Forward 5’-CCACACGTGGCCGGTCAGCA- 3’
	Reverse 5’-GTCCTGACCCTCTTGGGACGT-3’
S3247X (for sequencing)	Forward 5’-TGAAGCTTCCACTCATGCCG-3’
	Reverse 5’-ATGAAGCTTGTCCACGCGGA-3’
S3247X (for Taqman genotyping)	Forward 5’- CCAGAAACCATCGTGGATCTG-3’
	Reverse 5’-TGCCTGATTGTCTGGAGCG- 3’
	Wild type: FAM 5’-CAGTCAAGGCACGG-3’ MGB/BHQ
	Mutant: VIC 5’-AGCAGTAAAGGCACG-3’ MGB/BHQ

The amplicon was digested with *Hin1II* (Thermo Fisher Scientific, Waltham, MA, USA) for R501X, *AdeI* (Thermo Fisher Scientific Waltham, MA, USA) for 2282del4, and *BssSI* (NEB, Ipswich, MA, USA) for R2447X. The digested PCR fragments were run on 2% agarose gel. The S3247X mutations were screened using ABI 7500 (96-well) Fast Real-Time PCR systems. Fifty samples were randomly selected and sequenced for confirmation of the genotyping results. The sequencing reaction was performed by Big Dye Terminator cycle sequencing kit (version 3.2) using the ABI Prism 310 sequence detection system (Foster City, CA, USA).

### 2.3. Statistical analysis

SPSS 21.0 software (SPSS Inc., Chicago, IL, USA) was used for data analysis. Frequencies and percentages were used to describe categorical variables, and the comparisons of variables between groups were performed by chi-square test. The numerical variables were described as median (interquartile range) due to nonnormal distribution, comparisons between the groups in regard to numerical variables were obtained by Kruskal–Wallis test, and comparisons for categorical variables were established via χ2 test. A P-value <0.05 was considered statistically significant.

## 3. Results

Four hundred and sixty-six patients [67% male, 1 (0.7–2.8) years] and 128 healthy controls [59% male, 2.4 (1.4–3.5) years)] were included in this study. Only 2% of patients with AD were classified as having severe AD. One hundred and thirty-two patients (28%) had IgE-mediated FA without AD, 273 (59%) had FA and AD, and 61 (13%) had AD without FA. The age of onset for AD did not differ between patients with and without FA (p > 0.05), but the age of participants was different between groups (p < 0.001). The characteristic features of the patients and healthy controls are presented in Table 2.

**Table 2:** Demographic and clinical features of the study participants.

	Allergic Disease			HealthyControlsn = 128	P*	P†
	Food Allergyn = 132	Food Allergy &Atopic Dermatitisn = 273	AtopicDermatitisn = 61			
Current Age‡ (years)	2 (1-5.5)	1.0 (0.6––1.6)	2.0 (0.8–4.8)	2.4 (1.4–3.5)	<0.001	<0.001
Sex (male) n, (%)	86 (65.2)	191 (70.0)	37 (60.7)	75 (58.6)	NS	NS
Age of onset of AD‡(years)	–	0.3 (0.2–0.5)	0.3 (0.1–1.6)	-	<0.001	–
Eosinophil (%)‡	3.8 (2.3–7.2)	5.9 (3.3–9.1)	4.7 (3.0–7.3)	1.8 (1.2–3.0)	<0.001	<0.001
Eosinophil number‡ (/µL)	400 (200–700)	600 (327.5–1083.2)	400 (200–700)	193 (100–300)	<0.001	<0.001
Total IgE‡ (kU/L)	94.8 (40.5–271.3)	86.0 (32.3–272.8)	40.2 (8.5–188)	12.7 (5.0–38)	0.002	<0.001
Asthma, n (%)	62 (47.7)	70 (25.6)	7 (11.9)	-	<0.001	-
Allergic rhinitis, n (%)	27 (20.6)	29 (10.6)	10 (16.9)	-	0.022	-
Family hx of atopic disease, n (%)	43 (34.4)	110 (40.6)	24 (41.4)	21 (20)	NS	0.002
Cow’s milk allergy, n (%)	79 (60.8)	159 (59.1)	-	-	NS	-
Hen’s egg allergy, n (%)	53 (41.1)	230 (84.9)	-	-	<0.001	-
Tree nuts-peanut allergy, n (%)	36 (28.3)	85 (31.6)	-	-	NS	-
Multiple food allergy, n (%)	54 (41.5)	167 (61.6)	-	-	<0.001	-
Anaphylaxis in the hx or during OFC test	74 (56.1)	72 (26.4)	-	-	<0.001	-

*P: Comparison between the patients with allergic disease †P: comparison of the four groups of patients with and without allergic disease. ‡Median (interquartile range) OFC: Oral Food Challenge NS: Non significant p > 0.05.

According to genotyping results, two patients from FA group were heterozygous carriers of R501X, although none of the controls carried this mutation (Table 3). Three patients in the FA with AD group and one of the healthy children were heterozygous carriers of wild-type 2282del4. Neither patients nor controls carried the other two FLG mutations, R2447X and S3247X *.* We could not determine any combined mutation in the heterozygous mutation carriers. Table 3 shows the genetic analysis summary for study participants. Clinical features of the heterozygous carriers are given in Table 4.

**Table 3:** Summary of the genetic analysis of the study participants.

	Methods used	Food allergy	Food allergy +Atopic dermatitis	Atopic dermatitis	Healthy controls
R501X	PCR-RFLP n = 588 DNA sequencing n =38	Wild n = 129 Heterozygous n =2	Wild n = 268 Heterozygous : -	Wild n = 61 Heterozygous : -	Wild n = 128 Heterozygous : -
2282del4	PCR-RFLP n = 582 DNA sequencing n = 20	Wild n = 130 Heterozygous : -	Wild n = 268 Heterozygous n = 3	Wild n = 61 Heterozygous : -	Wild n = 119 Heterozygous n:1
R2447X	PCR-RFLP n = 588 DNA sequencing n = 32	Wild n = 129 Heterozygous : -	Wild n = 272 Heterozygous : -	Wild n = 61 Heterozygous : -	Wild n = 123 Heterozygous : -
S3247X	RT-PCR n = 522 DNA sequencing n = 81	Wild n = 131 Heterozygous : -	Wild n = 269 Heterozygous : -	Wild n = 54 Heterozygous : -	Wild n = 117 Heterozygous : -

**Table 4:** Features of the patients with FA and/or AD with heterozygous mutations for frequently encountered variants of filaggrin gene.

Patient No	Polymorphism	Sex	Age (year)	Type of food allergy	ConcurrentAD	Aeroallergen sensitization	Asthma	Food induced Anaphylaxis
1	R501X	Male	16	CM, hazelnut	-	Grass pollen	+	-
2	R501X	Female	2.4	Hazelnut	-	-	-	+
3	2282del4	Male	1	Hen’s egg	+	-	+	-
4	2282del4	Male	1	Hen’s egg	+	-	-	-
5	2282del4	Male	0.8	Hen’s egg, hazelnut	+	-	-	-

Although R501X, 2282del4, R2447X, and S3247X mutations are very common in European populations, according to our results, these FLG mutations are rarely found in Turkish individuals. Due to the low frequency of these mutations, no associations were shown between these mutations and FA and/or AD.

## 4. Discussion

In this study we revealed that the frequency of four common *FLG* LOF mutations was low or absent in a Turkish population and that these mutations were not a risk factor for FA and/or AD development in this population, although R501X, 2282del4, R2447X, and S3247X mutations are very common in European populations.

LOF mutations in the *FLG* gene are a major risk factor for allergic diseases such as AD, FA, allergic rhinitis, and asthma and are generally associated with the natural development of atopic march [5,21]. The association of changes in FLG with the abovementioned diseases was initially described in patients with ichthyosis vulgaris by Smith et al. in 2006 [20]. In that study, LOF mutations of R501X and 2282del4 were significantly associated with ichthyosis vulgaris in Irish, Scottish, and European American families [20].

In the years since, many population studies have been performed showing the importance of FLG mutations in atopic diseases, and population-specific LOF mutations have been confirmed by many studies [1,6,20,22]. According to these studies, 49 truncating mutations in the FLG gene have been identified as European-specific and Asian-specific mutations [23]. The most common mutations, R501X, 2282del4, R2447X, and S3247X existed with a frequency of 7%–10% in a white European population [22].

Population studies to date have primarily focused on the relationship between FLG mutations and AD; the relevance of atopic sensitization, allergic rhinitis, and asthma on FLG mutations has also been investigated [5]. Studies have shown that mutations of R501X and/or 2282del4 and, to a lesser extent, R2447X and/or S3247X were associated with early-onset AD [1,3,6,19].

The association between FLG-LOF mutations and asthma has been determined in many studies [24–26]. Although FLG-LOF mutations were an independent risk factor for asthma development in some studies, these mutations have a higher impact as risk factors for asthma development among AD patients [25,27–29]. The risk of developing asthma increased more than 2.5-fold (OR, 2.64; 95% CI, 1.76–4.00) in school children with AD compared to those without AD [26]. In addition, these LOF mutations in *FLG* were identified as risk factors for FA and concurrent AD with a progression into asthma [9,22]. However, contradictory results have been shown in studies investigating the function of FLG mutations in FA. Studies regarding FLG mutations and various allergens have supported the association of food sensitization and allergy, including peanut, egg, and cow’s milk, with FLG mutations. Brown et al. showed an association between peanut allergy and FLG-LOF mutations in children and adult patients from three different populations. Statistical significance was also obtained in the case of peanut allergy coexisting with AD [11]. In a birth cohort study, the association of peanut allergy with FLG mutations was found in children; a FLG-LOF mutation carrier had an increased risk for developing peanut sensitization and allergy when exposed to peanut in the first year of life [30]. FLG-LOF mutations were associated with food allergy and food allergen sensitization as determined by an allergen panel including egg, milk, soya, wheat, cod, and peanut [9]. Meanwhile, these significant results were usually obtained if the studied population had an AD background [9,10]. In contrast with these studies, LOF mutations were not associated with egg sensitization and egg allergy in the Melbourne cohort or with peanut sensitization and cow’s milk allergy in some other studies [8,31].

The most common FLG mutations (R501X and 2282del4) and the less common mutations in European populations (R2447X and S3247X) were rarely detected or not detected in Asian populations, and different mutations have been described for these populations. Asian-specific FLG mutations increase AD and AD with concurrent FA, but there was no association with asthma [32]. In Croatian patients with allergic diseases including atopic dermatitis, allergic rhinitis, asthma, and allergic contact dermatitis, low frequencies of FLG null mutations (R501X, 2282del4, R2447X, and S3247X) were detected; however, this study showed an association of FLG null mutations with other skin disorders [33].

Although R501X and 2282del4 FLG mutations are common in Europe, surprisingly, the studies including Italian populations revealed no significant association with AD. In these studies, three new specific mutations were identified by sequence analysis; however, these new variants were not associated with AD [34]. Paralleling the Italian study, a study including Egyptian patients showed no association of R501X and 2282del4 mutations with AD [35]. To our knowledge, the association between LOF mutations and AD in a Turkish population was investigated in only one prior study, and no association was found between the R501X mutation and Turkish children with AD [36].

The prevalence of AD varies greatly worldwide, by population, and it ranges from 0.9% to 20%. The prevalence of severe AD is much lower and is estimated at 1%–2% [37]. In the current study the AD group consisted of patients with mostly mild and moderate AD, and the ratio of severe AD was 2%. Because the study groups included few patients with severe AD, it may have been possible to obtain a higher frequency of LOF mutations, especially among severe AD groups.

The difference between the ages of patients and healthy controls may be a limitation of this study, but the frequency of LOF mutations was very low in both groups and there were no homozygous mutations in any of the participants. Additionally, although the onset of allergy and asthma symptoms may be related to the age of the patient, the frequency of LOF mutations in DNA cannot change over time. The method of food allergy diagnosis may also be a limitation of this study, as the gold standard is an oral food challenge test [38]. However, a consistent clear-cut history of allergic symptoms occurring after ingestion of a specific culprit food together with the presence of specific IgE has been one of the preferred methods for food allergy diagnosis in patients in certain respected publications [39]. Additionally, it is obvious that a clear-cut history with the culprit food together with specific IgE certainly increases the likelihood of an accurate diagnosis of FA, when compared to either self-reported history or food-specific IgE alone [38]. In conclusion, the results of this study show that FLG mutations particularly R501X, 2282del4, R2447X, and S3247X do not confer a risk for the development of FA and/or AD in a Turkish population. In addition to environmental factors and the influence of differential exposure to UV, a population-specific genetic background may be important when considering the association between FLG mutations and allergic diseases. Different FLG mutations, diversity in frequency, and varying relationships with allergic diseases among populations may express the influence of differential exposure to UV, with respect to geographic location. However, further studies are needed.

## Informed consent

This study was approved by the ethics committee of the Hacettepe University Medical Faculty (GO 13/100), and written informed consent was obtained from the patients, healthy controls, and their parents.
